# Evaluation of Common Type 2 Diabetes Risk Variants in a South Asian Population of Sri Lankan Descent

**DOI:** 10.1371/journal.pone.0098608

**Published:** 2014-06-13

**Authors:** Neelam Hassanali, N. Maneka G. De Silva, Neil Robertson, N. William Rayner, Amy Barrett, Amanda J. Bennett, Christopher J. Groves, David R. Matthews, Prasad Katulanda, Timothy M. Frayling, Mark I. McCarthy

**Affiliations:** 1 Oxford Centre for Diabetes, Endocrinology & Metabolism, University of Oxford, Churchill Hospital, Oxford, United Kingdom; 2 Genetics of Complex Traits, University of Exeter Medical School, Exeter, United Kingdom; 3 Wellcome Trust Centre for Human Genetics, University of Oxford, Oxford, United Kingdom; 4 Harris Manchester College, University of Oxford, Oxford, United Kingdom; 5 Department of Clinical Medicine, Faculty of Medicine, University of Colombo, Colombo, Sri Lanka; 6 Oxford National Institute for Health Research Biomedical Research Centre, Churchill Hospital, Oxford, United Kingdom; Sanjay Gandhi Medical Institute, India

## Abstract

**Introduction:**

Most studies seeking common variant associations with type 2 diabetes (T2D) have focused on individuals of European ancestry. These discoveries need to be evaluated in other major ancestral groups, to understand ethnic differences in predisposition, and establish whether these contribute to variation in T2D prevalence and presentation. This study aims to establish whether common variants conferring T2D-risk in Europeans contribute to T2D-susceptibility in the South Asian population of Sri Lanka.

**Methodology:**

Lead single nucleotide polymorphism (SNPs) at 37 T2D-risk loci attaining genome-wide significance in Europeans were genotyped in 878 T2D cases and 1523 normoglycaemic controls from Sri Lanka. Association testing was performed by logistic regression adjusting for age and sex and by the Cochran-Mantel-Haenszel test after stratifying according to self-identified ethnolinguistic subgroup. A weighted genetic risk score was generated to examine the combined effect of these SNPs on T2D-risk in the Sri Lankan population.

**Results:**

Of the 36 SNPs passing quality control, sixteen showed nominal (p<0.05) association in Sri Lankan samples, fifteen of those directionally-consistent with the original signal. Overall, these association findings were robust to analyses that accounted for membership of ethnolinguistic subgroups. Overall, the odds ratios for 31 of the 36 SNPs were directionally-consistent with those observed in Europeans (p = 3.2×10^−6^). Allelic odds ratios and risk allele frequencies in Sri Lankan subjects were not systematically different to those reported in Europeans. Genetic risk score and risk of T2D were strongly related in Sri Lankans (per allele OR 1.10 [95%CI 1.08–1.13], p = 1.2×10^−17^).

**Conclusion:**

Our data indicate that most T2D-risk variants identified in Europeans have similar effects in South Asians from Sri Lanka, and that systematic difference in common variant associations are unlikely to explain inter-ethnic differences in prevalence or presentation of T2D.

## Introduction

Type 2 diabetes (T2D) is a major global health concern that is currently estimated to affect 336 million people worldwide [1]. It is widely accepted that T2D is a complex disorder and individual risk reflects the influence of environmental factors on a background of genetic predisposition. Over the past three decades the prevalence of T2D in South Asians has shown a particularly dramatic increase [2], [3], prompted by profound changes in socioeconomic factors and lifestyle. Compared to European counterparts, South Asians tend to be diagnosed with diabetes earlier, with a lower BMI and display a more rapid decline in glycaemic control over time [3]. The increased prevalence of T2D extends to South Asian groups living outside their native countries, and this suggests that there may also be an underlying biological predisposition in addition to environmental and lifestyle factors [2], [4].

The advent of large scale genome-wide association studies (GWAS) has led to the identification of over 70 genetic loci that contribute to T2D risk [5]–[17]. Most of the early studies were conducted in Europeans, but increasingly, similar approaches are being deployed in samples of South Asian, East Asian and African origin [11], [13], [18]–[22]. These studies have revealed novel signals [4], [13], [23], but have also shown appreciable overlap with associations first discovered in European groups [19]–[22].

Here, we extend these studies to South Asians from the island of Sri Lanka. As elsewhere in South Asia, the incidence of T2D is increasing and it is predicted that by 2030 approximately 14% of the adult population will have the condition, many of them undiagnosed [1]. Relatively little is known about the genetic predisposition of T2D in this country. In this study, we determined whether a set of T2D-risk variants reaching genome-wide significance in Europeans carry the same disease risk in South Asians from Sri Lanka.

## Materials and Methodology

### Study Samples

Cases and controls were ascertained from two independent collections of South Asian subjects from Sri Lanka. T2D cases (n = 1001, 44% male) were recruits to the Sri Lankan Young Diabetes Study (SLYDS), consecutively ascertained from private and government diabetes clinics [24]. Age of diabetes diagnosis was between 16–40 years and all participants were under the age of 45 years at recruitment. Of the 1001, 965 had DNA samples available for genotyping. Within these individuals, T2D status was defined if Glutamic Acid Decarboxylase Autoantibodies (GADA) titre was ≤14 units/ml, and if the interval between diagnosis and the initiation of insulin therapy was at least six months [24]. Twenty-nine samples were excluded due to missing GADA data, 48 for having insulin treatment within 6 months of diagnosis and 10 individuals for a positive diagnosis of mitochondrial diabetes (mt3243 A>G;), leaving 878 T2D cases available for inclusion [24].

Control subjects were participants in the Sri Lankan Diabetes Cardiovascular Study (SLDCS), a cross-sectional epidemiological study that used a multi-stage random cluster sampling technique to recruit 4388 subjects across seven Sri Lankan provinces [25]. DNA collection was initiated partway through the study and DNA samples were available for 1769 subjects. Of these, 1523 individuals who were confirmed as normoglycaemic based on oral glucose tolerance data (interpreted according to then-current ADA and WHO criteria), and under the age of 80 years, were included in this study [26].

At the time of recruitment, participants in both studies were classified according to the major ethnolinguistic and religious groups in Sri Lanka, using categories specified, for example, in the national Census, and based on a combination of spoken language, religion/cultural identification and surname [27]. The majority were Sinhalese (86%) or Tamil (5.4%), with the rest categorised as Muslim (8.3%) or Burgher (0.26%) or having other designations (0.04%).

Participant collection was approved by the Ethical Review Committee of the University of Colombo. All participants provided informed written consent [24], [25].

### Genotyping and quality control

We genotyped the lead single nucleotide polymorphisms (SNPs) at 37 T2D-risk loci that had reached genome-wide significance in Europeans from studies published as of mid-2010 [28], [29]. We genotyped 2401 individuals (878 cases, 1523 controls) using Applied Biosystems TaqMan SNP genotyping assays on an Applied Biosystems 7900HT system. Seventy-four samples with a low (<80%) overall call rate were removed from further analysis leaving 2327 individuals (830 cases, 1497 controls) for final analysis. The average genotyping call rate for these 2327 individuals was 97%.

### Statistical Analysis

All 37 SNPs were in Hardy Weinberg equilibrium (HWE) except for the SNP rs2237892 in the *KCNQ1* locus (p<0.001 in controls) which was removed from subsequent analyses. First, we used logistic regression to assess the association between each individual SNP and T2D status assuming a log additive model. All associations were adjusted for age and sex. In addition we reanalysed the case-control data after stratifying for self-identified ethnic subgroup using the Cochran–Mantel–Haenszel (CMH) test. The individual SNP association analyses were undertaken in PLINK v1.07 [30], [31].

Next we tested the significance of genetic risk scores (GRS) that combine information from all 36 T2D associated SNPs using logistic regression. The SNPs were coded as 0,1 or 2 corresponding to the number of T2Ds risk increasing alleles in Europeans, except for the X chromosome SNP rs5945326 at *DUSP*9 where male genotypes were coded as 0 or 1, and female genotypes as 0, 0.5 or 1 (to reflect random X inactivation). These analyses were performed using Stata/SE version 10.1 for Windows (StataCorp, Brownsville, TX). To create the GRS, we used individuals with genotypes available from at least 29 of the 36 type 2 diabetes SNPs (i.e. 80% of the SNPs genotyped), and accounted for the varying effect sizes of each SNP using equation 1, where w is the natural log of the per allele type 2 diabetes odds ratio (OR) reported in Europeans.
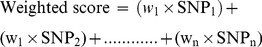
(1)We rescaled the weighted score from above to reflect the number of available SNPs using equation (2) as described previously [32].
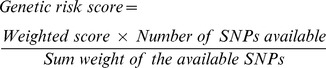
(2)We used this weighted GRS as the independent variable and T2D status as the dependent variable in logistic regression analyses. We also stratified individuals into quintiles of GRS.

To compare effect size estimates for Sri Lankan case-control samples with those observed in Europeans, we compiled odds ratio (OR) estimates for each locus for European case-control data from the literature [6], [7], [14]–[16], [29], [33]–[36] (Table S1). To minimise inflation of these estimates in initial genome-wide association discovery samples (the “winner's curse”), we used OR values from replication samples wherever possible.

### Power Calculations

Quanto was used to calculate power under assumptions of a log-additive model, a disease prevalence of 10%, and a significance threshold (α) of 0.05. Power was calculated for each individual SNP using allelic odds ratios (for European case-control comparisons) collated from the literature and effect allele frequencies from the CEU component of HapMap (Table S1) [37].

## Results

Individual SNP associations for the 36 SNPs in 830 T2D patients and 1497 controls are summarised in Table 1. Nominal associations with T2D (p≤0.05) were observed for 16 of the 36 SNPs tested when adjusted for age and sex. Given some case-control imbalance with respect to “self-identified” ethnolinguistic subgroups (e.g. Sinhala, Tamil), case-control analyses were repeated in stratified samples using the CMH test. The results obtained were broadly comparable with highly-correlated odds ratios (Table 1). In the CMH analysis, a total of 17 loci were nominally-associated with T2D, fourteen of them overlapping with the non-stratified analysis. Two loci (*IRS1* and *JAZF1*) were no longer associated (p<0.05) in the CMH test, but three others (*PROX1*, *PPARG* and *FTO*) became significant for the first time. Subsequent analyses were performed (unless otherwise stated) on the combined dataset.

**Table 1:** Individual SNP association results for T2D risk in the Sri Lankan case control samples.

Nearest Gene	CHR	SNP	Alleles	T2D Risk allele in Sri Lankans	T2D Risk allele in Europeans	RAF in Sri Lankans	Odds Ratio (95% Cl) adjusted for age and sex	p-value adjusted for age and sex	Odds ratio for CMH test (95% Cl) adjusted for age, sex	CMH test p-value adjusted for age, sex
*NOTCH2*	1	rs10923931	G/T	T	T	0.25	1.04 (0.90–1.20)	0.566	1.04 (0.90–1.20)	0.610
*PROX1*	1	rs340874	C/T	C	C	0.51	1.13 (1.00–1.29)	0.053	1.16 (1.02–1.31)	0.021
*BCL11A*	2	rs243021	G/A	A	A	0.49	1.23 (1.09–1.41)	1.1×10^−3^	1.25 (1.11–1.41)	3.6×10^−4^
*GCKR*	2	rs780094	C/T	C	C	0.20	0.87 (0.75–1.03)	0.110	0.91 (0.78–1.06)	0.21
*IRS1*	2	rs7578326	A/G	A	A	0.81	1.20 (1.03–1.41)	0.023	1.17 (1.00–1.37)	0.055
*THADA*	2	rs7578597	T/C	T	T	0.89	1.17 (0.96–1.44)	0.118	1.10 (0.91–1.35)	0.321
*ADAMTS9*	3	rs4607103	C/T	C	C	0.46	1.02 (0.89–1.15)	0.781	0.99 (0.87–1.11)	0.815
*ADCY5*	3	rs11708067	A/G	A	A	0.81	1.22 (1.04–1.45)	0.016	1.28 (1.09–1.50)	2.7×10^−3^
*IGF2BP2*	3	rs4402960	G/T	T	T	0.49	1.10 (0.97–1.26)	0.125	1.10 (0.97–1.25)	0.124
*PPARG*	3	rs1801282	C/G	C	C	0.91	1.21 (0.97–1.52)	0.093	1.30 (1.04–1.62)	0.021
*WFS1*	4	rs10010131	G/A	G	G	0.76	1.24 (1.07–1.45)	5.6×10^−3^	1.25 (1.07–1.45)	4.1×10^−3^
*ZBED3*	5	rs4457053	A/G	G	G	0.20	1.25 (1.08–1.46)	3.6×10^−3^	1.23 (1.06–1.43)	6.7×10^−3^
*CDKAL1*	6	rs10946398	T/C	C	C	0.24	1.09 (0.94–1.27)	0.240	1.07 (0.92–1.24)	0.390
*DGKB/TMEM195*	7	rs2191349	T/G	T	T	0.66	0.99 (0.86–1.12)	0.821	0.99 (0.87–1.13)	0.855
*GCK*	7	rs4607517	G/A	A	A	0.12	0.92 (0.75–1.13)	0.441	0.93 (0.76–1.13)	0.449
*JAZF1*	7	rs864745	C/T	T	T	0.79	1.17 (1.00–1.37)	0.049	1.14 (0.98–1.33)	0.082
*KLF14*	7	rs972283	G/A	G	G	0.63	1.01 (0.89–1.16)	0.834	1.03 (0.91–1.17)	0.633
*SLC30A8*	8	rs13266634	C/T	C	C	0.77	1.32 (1.14–1.55)	3.9×10^−4^	1.33 (1.14–1.55)	3.0×10^−4^
*TP53INP1*	8	rs896854	C/T	T	T	0.40	1.03 (0.90–1.17)	0.66	1.02 (0.90–1.16)	0.761
*CDKN2A/B*	9	rs10811661	T/C	T	T	0.85	1.34 (1.12–1.61)	1.2×10^−3^	1.32 (1.10–1.57)	2.0×10^−3^
*CHCHD9*	9	rs13292136	C/T	C	C	0.84	1.29 (1.08–1.54)	4.4×10^−3^	1.31 (1.10–1.56)	2.4×10^−3^
*CDC123/CAMK1D*	10	rs12779790	A/G	G	G	0.14	1.09 (0.91–1.31)	0.327	1.09 (0.91–1.30)	0.346
*HHEX/IDE*	10	rs1111875	C/T	C	C	0.36	1.14 (1.01–1.30)	0.048	1.17 (1.03–1.32)	0.017
*TCF7L2*	10	rs7903146	C/T	T	T	0.34	1.38 (1.21–1.59)	2.8×10^−6^	1.35 (1.19–1.54)	6.1×10^−6^
*CENTD2*	11	rs1552224	A/C	A	A	0.82	1.12 (0.95–1.33)	0.174	1.11 (0.95–1.31)	0.194
*KCNJ11*	11	rs5219	C/T	T	T	0.34	1.15 (1.01–1.31)	0.035	1.16 (1.02–1.31)	0.026
*KCNQ1*	11	rs231362	A/G	G	G	0.76	1.03 (0.89–1.19)	0.728	1.03 (0.90–1.20)	0.644
*MTNR1B*	11	rs10830963	C/G	G	G	0.84	1.12 (0.99–1.27)	0.067	1.10 (0.97–1.25)	0.132
*HMGA2*	12	rs1531343	G/C	C	C	0.19	1.23 (1.05–1.45)	8.8×10^−3^	1.22 (1.04–1.43)	0.013
*HNF1A*	12	rs7957197	T/A	T	T	0.96	0.94 (0.67–1.33)	0.735	1.02 (0.73–1.42)	0.907
*TSPAN8/LGR5*	12	rs7961581	T/C	C	C	0.35	0.87 (0.77–0.99)	0.049	0.84 (0.74–0.96)	0.012
*PRC1*	15	rs8042680	A/C	A	A	0.74	1.18 (1.03–1.37)	0.020	1.24 (1.08–1.43)	0.003
*ZFAND6*	15	rs11634397	G/A	G	G	0.53	1.00 (0.89–1.14)	0.933	1.00 (0.88–1.13)	0.996
*FTO*	16	rs9939609	A/T	A	A	0.36	1.11 (0.98–1.27)	0.089	1.14 (1.00–1.30)	0.044
*HNF1B(TCF2)*	17	rs757210	C/T	T	T	0.28	1.01 (0.88–1.16)	0.851	0.99 (0.87–1.14)	0.931
*DUSP9*	X	rs5945326	A/G	A	A	0.57	1.15 (1.03–1.29)	0.010	1.29 (1.14–1.47)	6.1×10^−5^

Chr: chromosome.

The strongest effects were seen at *TCF7L2* (OR 1.38 [95% CI 1.21–1.58], p = 2.8×10^−6^) and *SLC30A8* (OR 1.32 [95% CI 1.14–1.55], p = 3.9×10^−4^). At 15 of the 16 loci, the direction of effect was consistent with that reported in the original discovery study, the exception being rs7961581 (*TSPAN8/LGR5*: OR 0.87 [95% CI 0.77–0.99], p = 0.049). In all, 31 of the 36 SNPs tested showed evidence of association that was directionally consistent with previous studies in Europeans (i.e. the same allele increases T2D risk in the two populations) (binomial p = 3.2×10^−6^).

**Figure 1:**
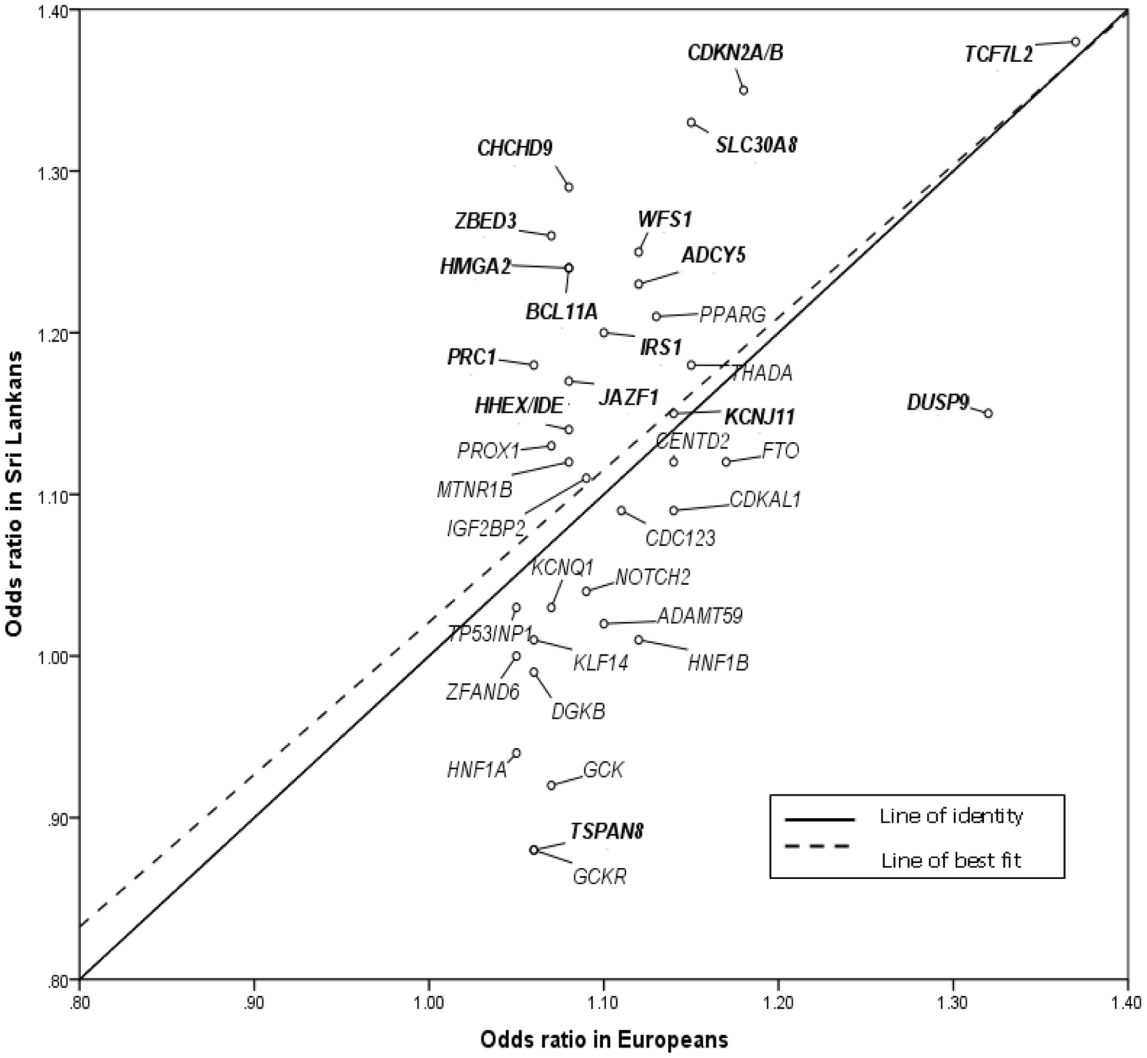
Comparison of allelic ORs between South Asians from Sri Lanka and Europeans. Loci labelled in bold are variants that showed nominal significance (p<0.05) in Sri Lankan subjects. European ORs used were derived from previously reported studies (Table S1).

**Figure 2:**
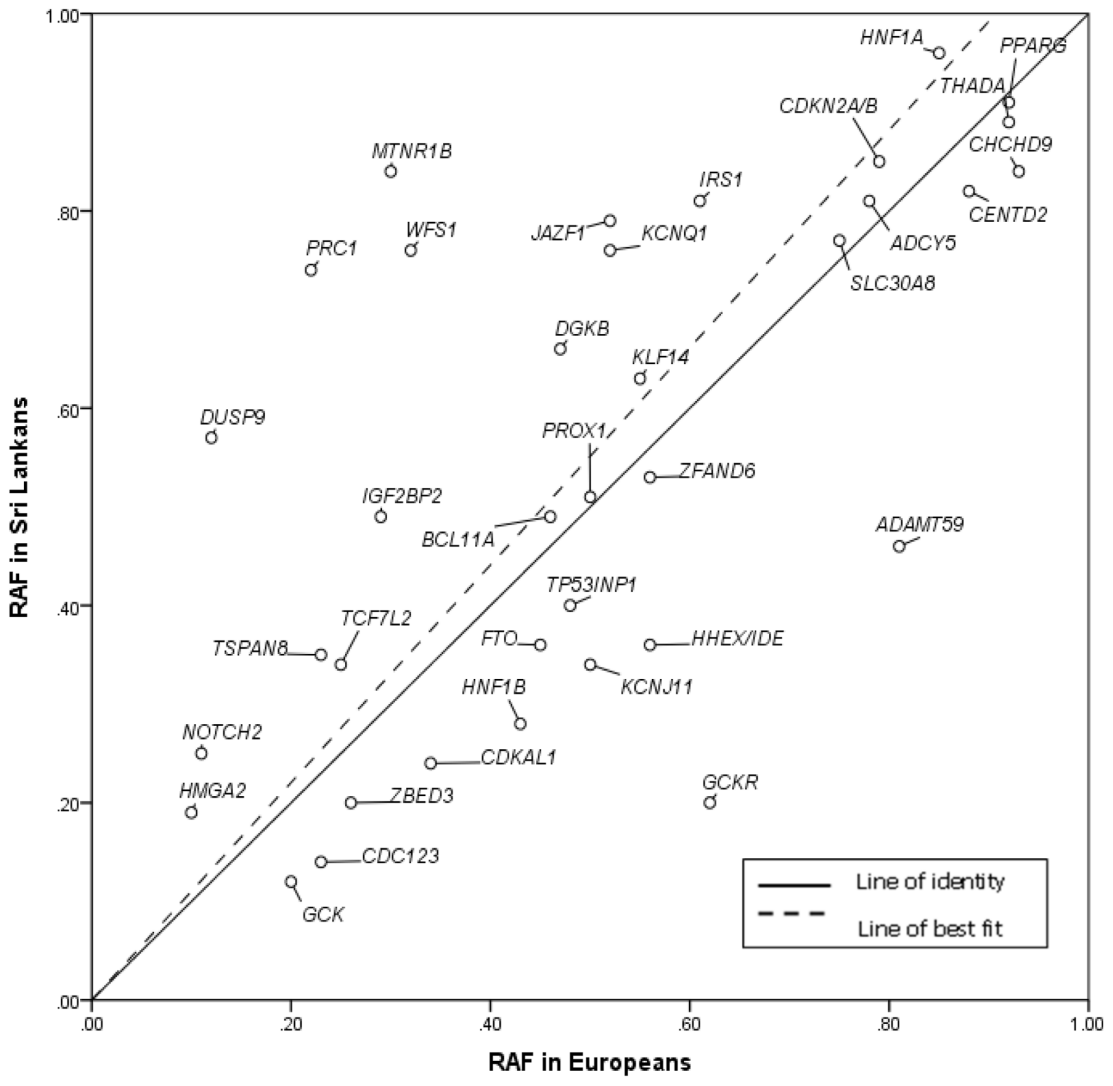
Comparison of risk allele frequencies (RAF) between South Asians from Sri Lanka samples included in this study (cases and controls combined) and previously reported RAF for Europeans (from HapMap).

Given the substantial differences in prevalence and presentation of T2D between European and South Asian populations, we sought evidence for consistent differences in effect size or allele frequency between the present study and previous reports from European studies (see methods). We found significant correlations between Sri Lankan and European samples for both the allelic odds ratio point estimates (Figure 1; r = 0.50, p = 1.8×10^−3^) and risk allele frequencies (Figure 2: r = 0.64, p = 2.3×10^−5^) but no suggestion of systematic differences in either. Allelic OR point estimates were greater in Sri Lankans than Europeans at 19 of 36 loci, and risk allele frequencies at 20 of 36.

**Figure 3:**
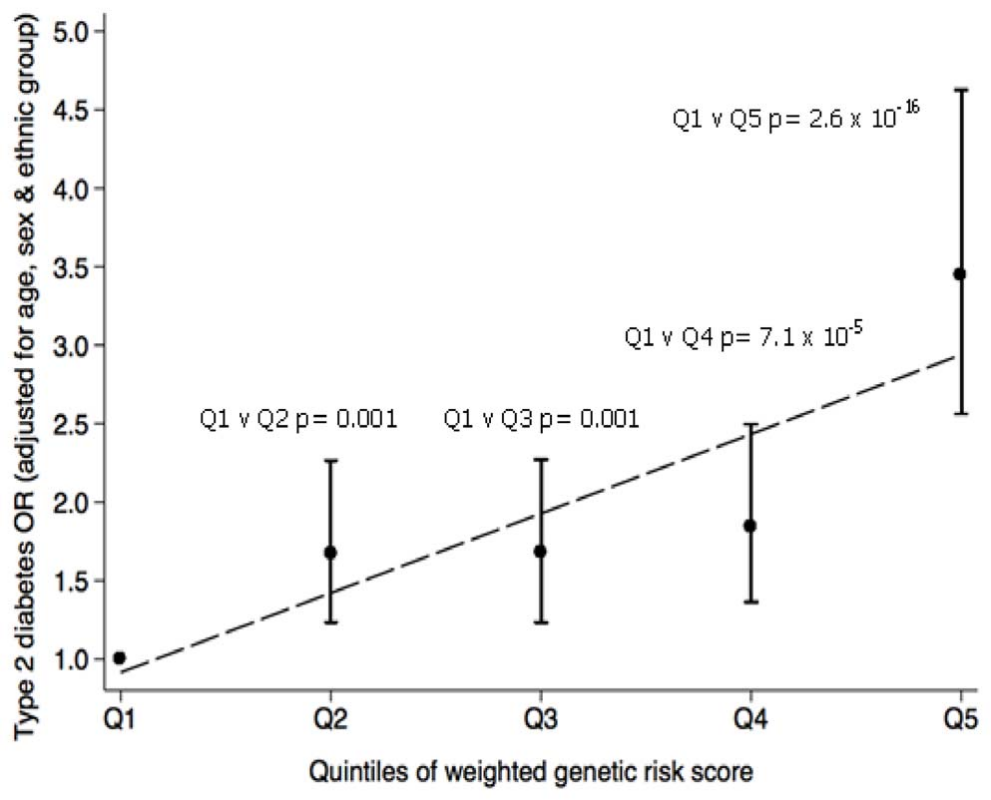
The combined impact of the 36 T2D-associated SNPs on T2D risk in T2D cases and controls of South Asian origin from Sri Lanka. Subjects were grouped into quintiles of the weighted genetic risk score. Circles represent the T2D odds ratio (adjusted for age, sex and ethnic group) when comparing each quintile group to the group in the lowest quintile (Q1). The capped lines represent the 95% CI of the T2D odds ratios.

As expected, individuals carrying greater numbers of (weighted) T2D risk increasing alleles had increased T2D risk (Figure 3), with an allelic OR of 1.10 (95%CI: 1.08–1.13, p = 1.2×10^−17^) per unit of the weighted genetic risk score. Individuals in the highest quintile of the genetic risk score had more than three-fold higher odds of T2D (3.44 [95%CI: 2.56–4.63] p = 2.6×10^−16^) when compared to individuals in the lowest quintile.

## Discussion

In this study, we have shown that established T2D-risk variants, most of them first identified in European samples, show strong enrichment of association in T2D cases and controls of South Asian origin from Sri Lanka. This pattern of enrichment, along with the absence of any systematic difference in risk-allele frequency or odds ratios between Sri Lankan and European samples has several important corollaries.

Firstly, these data provide further evidence for the transethnic consistency in allelic patterns of association for T2D, building on similar findings seen in a variety of ethnic groups including other samples of South Asian origin [17], [19]–[22], [38]. These patterns of transethnic consistency are consistent with a model in which the (often unknown) casual variants driving these association signals are also themselves common, a model which is also increasingly supported by fine mapping data [17], [39]. However, definitive confirmation of this model will require comprehensive identification of variants in these regions (e.g. via genome sequencing studies that are ongoing) such that the contribution of variants of all frequencies to disease predisposition can be directly tested.

Secondly, despite differences in both the prevalence and presentation of T2D between Sri Lanka and Europe, we observed no systematic differences in either risk allele frequency or effect size. We conclude therefore, that these ethnic differences in epidemiological and physiological patterns cannot be attributed to differences in common variant predisposition.

Though the general patterns are clear, the relatively modest sample sizes available in this study limit the inferences that can be made at any individual locus. None of the variants tested reached stringent genome-wide significance, and only about half of the loci reached nominal significance (i.e. p≤0.05). The patterns of association seen even amongst those variants not reaching nominal significance (from the twenty SNP associations with p>0.05, fifteen are directionally consistent with the associations reported in Europeans) indicate that many of these are likely to be false-negatives reflecting the limited power of our study. Many of these loci have very modest odds ratios and would have required much larger sample sizes to be detected than were available to us. Indeed, amongst the 15 loci with no formal evidence (p>0.05) of association in our study, but displaying directional consistency with data from Europeans, are several that show evidence of association in other South Asian case-control studies. For example, variants at the *GCKR* and *CDC123* loci were not associated with T2D in the present study, but have strong associations in far larger meta-analyses of South Asian samples [13].

In summary, we have shown that common T2D risk variants identified in Europeans have a similar genetic risk in Sri Lankans, adding further to the evidence that South Asians and Europeans share many overlapping common variants which contribute to T2D risk.

## Supporting Information

Table S1 **Summary of the reported allele frequency and odds ratio in Europeans for the T2D SNPs investigated.**
(DOCX)
