# Supplementary material for: Evaluation of Common Type 2 Diabetes Risk Variants in a South Asian Population of Sri Lankan Descent
Source: PLoS One. 2014 Jun 13;9(6):e98608. doi: 10.1371/journal.pone.0098608 (PMC4057178; doi:10.1371/journal.pone.0098608)
Supplement: Table S1 — Summary of the reported allele frequency and odds ratio in Europeans for the T2D SNPs investigated. (DOCX) [file pone.0098608.s001.docx]

***Table S1: Summary of the reported allele frequency and odds ratio in Europeans for the T2D SNPs investigated***

| **Locus** | **Chr.** | **SNP** | **Power** | **Effect allele frequency (HapMap/CEU)** | **Odds ratio European studies** | **Reference for European Odds ratio** |
| --- | --- | --- | --- | --- | --- | --- |
| *NOTCH2* | 1 | rs10923931 | 0.15 | 0.11 | 1.09 | Zeggini et al, 2008 (2nd stage replication) |
| *PROX1* | 1 | rs340874 | 0.20 | 0.5 | 1.07 | Dupuis *et al*, 2010 (stage 1 replication data) |
| *BCL11A* | 2 | rs243021 | 0.24 | 0.46 | 1.08 | Voight *et al*, 2010 (2nd stage replication) |
| *GCKR* | 2 | rs780094 | 0.15 | 0.62 | 1.06 | Dupuis *et al*, 2010 (stage 1 replication data) |
| *IRS1* | 2 | rs2943641 | 0.33 | 0.61 | 1.1 | Voight et al, 2010 (2nd stage replication) |
| *THADA* | 2 | rs7578597 | 0.23 | 0.92 | 1.15 | Zeggini et al, 2008 (2nd stage replication) |
| *ADAMT59* | 3 | rs4607103 | 0.23 | 0.81 | 1.1 | Zeggini et al, 2008 (2nd stage replication) |
| *ADCY5* | 3 | rs11708067 | 0.33 | 0.78 | 1.12 | Dupuis *et al*, 2010 (stage 1 replication data) |
| *IGF2BP2* | 3 | rs4402960 | 0.25 | 0.29 | 1.09 | Zeggini et al, 2007 Replication Meta-analysis |
| *PPARG* | 3 | rs1801282 | 0.18 | 0.92 | 1.13 | Altshuler *et* al, 2000 |
| *WFS1* | 4 | rs1001013 | 0.41 | 0.32 | 1.12 | Sandhu *et al,* 2007 |
| *ZBED3* | 5 | rs4457053 | 0.16 | 0.26 | 1.07 | Voight *et al*, 2010 (2nd stage replication) |
| *CDKAL1* | 6 | rs10946398 | 0.53 | 0.34 | 1.14 | Zeggini et al, 2007 (stage 1 replication data) |
| *DGKB* | 7 | rs2191349 | 0.16 | 0.47 | 1.06 | Dupuis *et al*, 2010 (stage 1 replication data) |
| *GCK* | 7 | rs4607517 | 0.15 | 0.2 | 1.07 | Dupuis *et al*, 2010 (stage 1 replication data) |
| *JAZF1* | 7 | rs864745 | 0.24 | 0.52 | 1.08 | Zeggini et al, 2008 (2nd stage replication) |
| *KLF14* | 7 | rs972283 | 0.16 | 0.55 | 1.06 | Voight *et al*, 2010 (2nd stage replication) |
| *SLC30A8* | 8 | rs13266634 | 0.50 | 0.75 | 1.15 | Sladek *et al,*2007 |
| *TP53INP1* | 8 | rs896854 | 0.13 | 0.48 | 1.05 | Voight *et al*, 2010 (2nd stage replication) |
| *CDKN2A/B* | 9 | rs10811661 | 0.58 | 0.79 | 1.18 | Zeggini et al, 2007 (stage 1 replication data) |
| *CHCHD9* | 9 | rs13292136 | 0.10 | 0.93 | 1.08 | Voight *et al*, 2010 (2nd stage replication) |
| *CDC123* | 10 | rs12779790 | 0.31 | 0.23 | 1.11 | Zeggini et al, 2008 (2nd stage replication) |
| *HHEX/IDE* | 10 | rs1111875 | 0.24 | 0.56 | 1.08 | Sladek, 2007 |
| *TCF7L2* | 10 | rs7903146 | 1.00 | 0.25 | 1.37 | Grant *et al,* 2006 |
| *CENTD2* | 11 | rs1552224 | 0.27 | 0.88 | 1.14 | Voight *et al*, 2010 (2nd stage replication) |
| *KCNJ11* | 11 | rs5219 | 0.57 | 0.5 | 1.14 | Gloyn *et al,*2003 |
| *KCNQ1* | 11 | rs231362 | 0.20 | 0.52 | 1.07 | Voight *et al*, 2010 (2nd stage replication) |
| *MTNR1B* | 11 | rs10830963 | 0.22 | 0.3 | 1.08 | Voight *et al*, 2010 (2nd stage replication) |
| *HMGA2* | 12 | rs1531343 | 0.12 | 0.1 | 1.08 | Voight *et al*, 2010 (2nd stage replication) |
| *HNF1A* | 12 | rs7957197 | 0.09 | 0.85 | 1.05 | Voight *et al*, 2010 (2nd stage replication) |
| *TSPAN8* | 12 | rs7961581 | 0.13 | 0.23 | 1.06 | Zeggini et al, 2008 (2nd stage replication) |
| *PRC1* | 15 | rs8042680 | 0.12 | 0.22 | 1.06 | Voight *et al*, 2010 (2nd stage replication) |
| *ZFAND6* | 15 | rs11634397 | 0.12 | 0.56 | 1.05 | Voight *et al*, 2010 (2nd stage replication) |
| *FTO* | 16 | rs8050136 | 0.73 | 0.45 | 1.17 | Frayling 2007 |
| *HNF1B* | 17 | rs757210 | 0.45 | 0.43 | 1.12 | Winckler *et al,* 2007 |
| *DUSP9* | X | rs5945326 | 0.87 | 0.12 | 1.32 | Voight *et al*, 2010 (2nd stage replication) |

*****Power was calculated using Quanto assuming a disease prevalence of 10% and a significance threshold (α) of 0.05, effect allele frequencies from the CEU component of HapMap and allelic odds ratios from European populations using the sample size of 830 cases and 1497 controls.
